# Position statement of Hepatology Society, Dhaka, Bangladesh, on the management of acute variceal bleeding in a resource-limited setting

**DOI:** 10.1016/j.iliver.2022.11.004

**Published:** 2022-11-19

**Authors:** Tanvir Ahmad, Shahinul Alam, Saiful Islam, Golam Azam, Mahabubul Alam, SKM Nazmul Hasan, Golam Mustafa, Motahar Hossain, Md. Akmat Ali, Mobin Khan

**Affiliations:** a Kurmitola General Hospital, Dhaka, Bangladesh; b Department of Hepatology, BSMMU General Secretary, Hepatology Society, Dhaka, Bangladesh; c Department of Hepatology, BSMMU, Bangladesh; d Department of Gastrointestinal-Hepatobiliary and Pancreatic Disorders (GHPD), BIRDEM Hospital, Bangladesh; e Department of Hepatology, Shahid Syed Nazrul Islam Medical College, Kishoregonj, Bangladesh; f Square Hospital, Bangladesh; g Addin Medical College, Dhaka, Bangladesh

**Keywords:** Variceal bleeding, Resource-limited setting, Terlipressin, Cirrhosis, Primary care physician

## Abstract

Variceal bleeding is one of the important signs of decompensation in patients with cirrhosis of the liver. It is always a medical emergency and sometimes results in death. Every year many patients die due to acute bleeding worldwide. The outcome depends on bleeding and its complications as well as the severity of the underlying liver disease. Careful volume resuscitation, administration of antibiotics and vasoactive drugs, and early endoscopic therapy prevent rebleeding and death. People living in rural areas are first referred to a district hospital from the Upazila health complex for any medical emergency. So, commencing the resuscitation process as well as administration of the vasoactive drug (terlipressin) at the first attending hospital before being referred to a higher center will decrease the mortality in patients presenting with acute variceal bleeding.

## 1 Introduction

Cirrhosis of the liver is a chronic disease with high morbidity and mortality. A large number of patients die from it every year, making it the fifth-leading cause of adult deaths worldwide [1]. It is a heterogeneous chronic disorder that needs a multidisciplinary approach. It has two stages: compensated and decompensated cirrhosis of the liver [2,3]. These two stages are differentiated by the presence or absence of decompensating signs like ascites, variceal bleeding, and/or hepatic encephalopathy. In compensated cirrhosis, the median survival is about 12 years; on the other hand, the median survival in decompensated cirrhosis of the liver is only 1.8 years [4]. The Child–Turcotte–Pugh (CTP) scoring helps in classifying patients with cirrhosis. It is also being used in prognosis. Patients who fall into CTP-A are compensated, whereas those who are in the CTP-B/C are mostly decompensated [5].

## 2 Portal hypertension

Portal hypertension plays a major role in developing complications in cirrhosis of the liver. Measurement of portal pressure helps in identifying the development of complications of cirrhosis. The portal pressure can be measured indirectly by determining the hepatic venous pressure gradient (HVPG) which is the difference between the wedged and the free hepatic venous pressures. It showed better results than liver histology [6]. It is termed portal hypertension when the HVPG is more than 5 mm Hg. The normal HVPG range is about 3–5 mm Hg. It becomes clinically significant when it crosses ≥10 mmHg. At this point, there is a high risk of developing esophageal or gastric varices, signs of decompensation, liver failure following surgery, and hepatocellular carcinoma [5].

## 3 Epidemiology and associated conditions

**Fig. 1:**
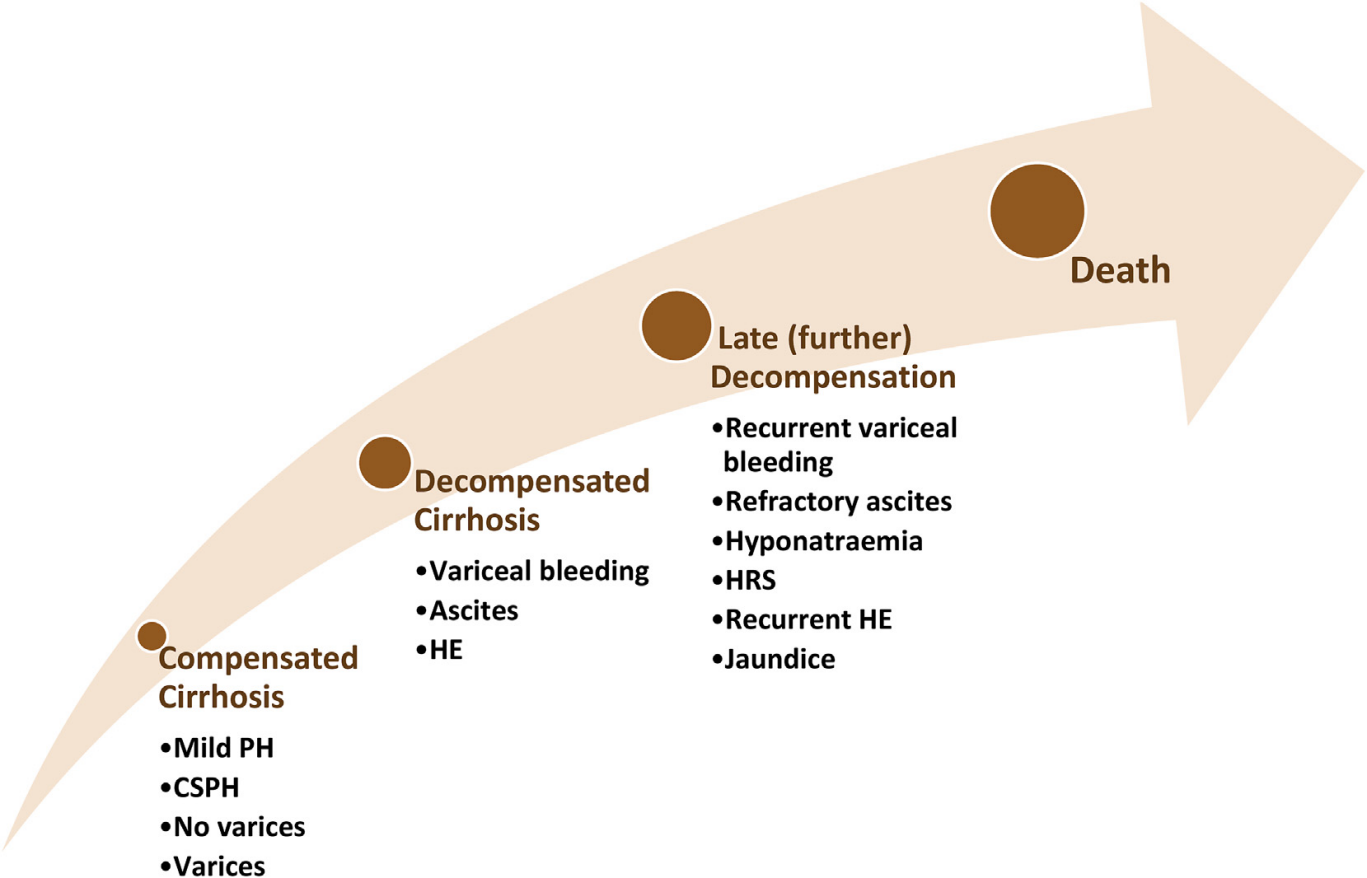
Natural history of cirrhosis of the liver. There are two stages, namely, compensated and decompensated cirrhosis. The main signs of decompensation are ascites, variceal bleeding, and hepatic encephalopathy. The compensated stage of cirrhosis is the longest period. Patients with compensated cirrhosis may have mild portal hypertension (mild PH) or clinically significant portal hypertension (CSPH). In CSPH, patients may have varices or not. But patients with CSPH are at high risk of developing decompensation. The decompensated stage may rapidly progress to further decompensation. At this stage, patients may develop renal failure (hepatorenal syndrome) and liver failure (encephalopathy and jaundice), leading to high mortality [3].

About 50% of cirrhotic patients may present with varices. It depends on the stages. Gastroesophageal varices may present at least 30%–40% in compensated cirrhosis; on the other hand, up to 85% of the decompensated cirrhotic patients may present with it [7]. Depending on the severity of the liver disease (Fig. 1), bleeding from gastroesophageal varices occurs about 10%–15% per year [8]. To assess the outcome of treatment, 6-week mortality has been considered the primary endpoint and which is about 15%–25% [9].

## 4 Management of acute variceal bleeding

When a patient presents with acute variceal bleeding, he/she should be considered decompensated. Overall five-year mortality is very different. It depends on the mode of presentation. The mortality is about 20% if the patients develop only variceal bleeding. On the other hand, mortality crosses over 80% when other decompensating signs such as ascites and hepatic encephalopathy are present along with bleeding [4].

### 4.1 Risk stratification

**Fig. 2:**
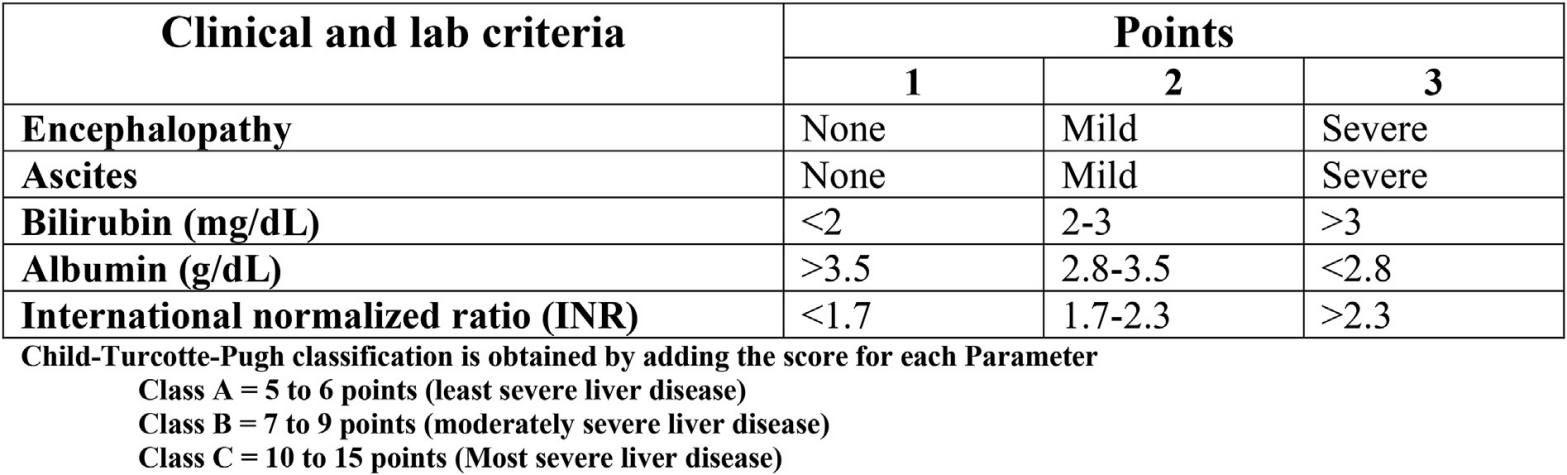
Child–Turcotte–Pugh classification.

Measurement of HVPG to stratify risk is very essential in a patient presenting with acute variceal bleeding. HVPG is a very good predictor of early rebleeding or even death when it increases to 20 mm Hg or more and if measured within 24 h of admission [10]. However, this is not available in Bangladesh. The variable used for calculating the CTP score is easily available and cheap. Alternatively, it can be used during the phase of acute variceal bleeding. According to various studies, a significant association was found between the CTP class and an HVPG. The studies showed that more than 80% of patients with CTP-C (Fig. 2) are having an HVPG ≥20 mm Hg [11].

### 4.2 Steps of management

Patients should be managed according to the following steps:

a. Pre-endoscopic management (resuscitation phase)
b. Endoscopic management
c. Post-endoscopic management

#### 4.2.1 Pre-endoscopic management

Acute variceal bleeding is a medical emergency. An initial assessment monitoring pulse, blood pressure, as well as respiratory rate, and protection of the airway and circulation is very essential in managing a patient with upper GI bleeding of any cause. Patients with altered levels of consciousness or those actively vomiting blood should be intubated before endoscopy.

##### 4.2.1.1 Volume replacement

After the initial assessment, volume replacement should be commenced promptly to prevent hypovolaemic shock. In an acute setting, fluids, such as colloids (albumin) or crystalloids (dextrose aqua/dextrose saline/normal saline), should be used. Starch (hydroxyethyl starch) should not be used for volume replacement [12].

##### 4.2.1.2 Blood transfusion

Blood transfusion should be started if the hemoglobin level reaches below 7 g/dL. In acute variceal bleeding, it is recommended to follow the “restrictive” packed red blood cell transfusion approach, which enormously decreases mortality. In a restrictive approach, blood is transfused to keep hemoglobin between 7 and 9 g/dL. In the ‘liberal” approach, packed red blood cell transfusion is initiated at a hemoglobin level of 9 g/dL and kept between 9 and 11 g/dL [5]. Various studies showed that liberal transfusion increased HVPG, risk of rebleeding, and mortality in patients in CTP stages A and B [13].

##### 4.2.1.3 Control of infection

Cirrhotic patients presenting with variceal bleeding are highly threatened by growing bacterial infections. In some randomized controlled trials, it was shown that initiating antibiotic prophylaxis decreased the rate of infection, rebleeding, and death [14]. So, it is recommended to start intravenous ceftriaxone at a dose of 1 g every day for a maximum of 7 days [5].

##### 4.2.1.4 Vasoactive agents

Commencing vasoactive drugs at an early period in patients with acute variceal bleeding lowers mortality and the requirement for blood transfusion [15]. So, before the diagnostic endoscopy, the vasoactive drugs should be given to all patients (if no contraindications) with variceal bleeding as early as possible along with antibiotics. All the drugs used to control variceal bleeding are administered in intravenous infusion. In our country, terlipressin and somatostatin are available. Somatostatin should be used in continuous IV infusion, whereas terlipressin can be given as a bolus.

**Somatostatin:**

Initial dose:
IV bolus 250 μg
Maintenance: Intravenous continuous infusion of 250–500 μg/h
Duration – 2–5 days.

**Terlipressin:**

Initial dose :
Initial 48 h: 2 mg intravenously every 4 h until control of bleeding
Maintenance: 1 mg intravenously every 4 h to prevent rebleeding
Duration: 2–5 days.

##### 4.2.1.5 Proton-pump inhibitor

It is evident that proton-pump inhibitor plays a major role in controlling nonvariceal bleeding such as bleeding from peptic ulcers. The role is unclear in variceal bleeding. Proton-pump inhibitor that is started prior to endoscopy should be stopped immediately after the procedure unless there is a strict indication to continue it. Long-term use also has been discouraged due to the chance of developing bacterial peritonitis in cirrhotic patients with ascites [16].

#### 4.2.2 Endoscopic management

It is recommended to start upper GI endoscopy within 12–24 h after initiating the resuscitation to find out the cause of bleeding and to give therapy if needed. The Previous study showed that about 30% of cirrhotic patients' causes of upper GI bleeding are due to nonvariceal causes [12]. If the first attending hospital starts the resuscitation at their center and then refers to a higher center for endoscopic management, both time and life will be saved.

## 5 Conclusion

The main goal of treatment in patients with variceal bleeding is to control bleeding, prevent early recurrence (within 5 days), and prevent 6-week mortality. Effective initiation of resuscitation and first dose of terlipressin at the first attending hospital will decrease mortality, improve the outcome, and help to initiate early endoscopic management at the tertiary-level hospital.

## Funding

The publishing will be supported by the Hepatology Society Dhaka Bangladesh.

## Author contributions

Tanvir Ahmad: Conception, design, draft preparation; Shahinul Alam: Conception, design, draft preparation; Saiful Islam: Conception, design; Golam Azam: Conception, design; Mahabubul Alam: Conception, design; SKM Nazmul Hasan: Conception, design; Golam Mustafa: Conception, design; Motahar Hossain: Conception, design; MD Akmat Ali: Conception, design; Abu Sayeed: Conception, design; Mobin Khan: Conception, design.

## Data Availability

As it is a position statement based on various guidelines, no data can be provided.

## Statement of intent

We have developed this statement based on the available evidence for clinical practice for the first attending hospital. We also declare that this statement may not inevitably ensure the best outcome in every situation.

## Objective

Providing evidence-based recommendations to physicians for managing acute variceal bleeding only in the resource-limited area is the primary objective of this statement. Therefore, this is not a complete overview of all aspects of variceal bleeding.

## Target population

This statement only applies to patients who present with acute variceal bleeding in the resource-limited center or where endoscopic management is unavailable.

## Target group/user

This position statement is proposed for the physicians who evaluate and manage patients with acute variceal bleeding in the first attending resource-limited hospital.

## Appendix A Supplementary data

Multimedia component 1 Algorithm of management of acute variceal bleeding in a resource-limited setting. Multimedia component 1
