# Supplementary material for: Position statement of Hepatology Society, Dhaka, Bangladesh, on the management of acute variceal bleeding in a resource-limited setting
Source: ILIVER. 2022 Nov 19;1(4):205–8. doi: 10.1016/j.iliver.2022.11.004 (PMC12212661; doi:10.1016/j.iliver.2022.11.004)
Supplement: Multimedia component 1 — Algorithm of management of acute variceal bleeding in a resource-limited setting. [file mmc1.docx]

# Algorithm of management of acute variceal bleeding in a resource-limited setting

**Patient with cirrhosis of liver presented with upper GI bleeding at the emergency**

**Initial assessment (Pulse, BP) protection of circulation and airway**

**Volume replacement**

**(Colloid or crystalloid)**

**Referred to higher center**

**IV Proton pump inhibitor**

**Inj. Terlipressin 2 mg IV stat**

**Inj. Ceftriaxone 1 gm IV stat**

**Restrictive blood transfusion**

**if haemoglobin < 7 gm/dl**
